# The anti-cancer efficacy of a novel phenothiazine derivative is independent of dopamine and serotonin receptor inhibition

**DOI:** 10.3389/fonc.2023.1295185

**Published:** 2023-10-16

**Authors:** Marion Vanneste, Anita Venzke, Soumitra Guin, Andrew J. Fuller, Andrew J. Jezewski, Sarah R. Beattie, Damian J. Krysan, Marvin J. Meyers, Michael D. Henry

**Affiliations:** ^1^ Department of Molecular Physiology and Biophysics, Carver College of Medicine, University of Iowa, Iowa City IA, United States; ^2^ Holden Comprehensive Cancer Center, University of Iowa, Iowa City, IA, United States; ^3^ Department of Chemistry, Saint Louis University, Saint Louis, MO, United States; ^4^ Department of Pediatrics, Carver College of Medicine, University of Iowa, Iowa City, IA, United States; ^5^ Department of Microbiology and Immunology, Carver College of Medicine, University of Iowa, Iowa City, IA, United States

**Keywords:** drug repurposing, phenothiazine, anti-cancer effect, blood-brain barrier, mitotic arrest, calmodulin, metastasis

## Abstract

**Introduction:**

An attractive, yet unrealized, goal in cancer therapy is repurposing psychiatric drugs that can readily penetrate the blood-brain barrier for the treatment of primary brain tumors and brain metastases. Phenothiazines (PTZs) have demonstrated anti-cancer properties through a variety of mechanisms. However, it remains unclear whether these effects are entirely separate from their activity as dopamine and serotonin receptor (DR/5-HTR) antagonists.

**Methods:**

In this study, we evaluated the anti-cancer efficacy of a novel PTZ analog, CWHM-974, that was shown to be 100-1000-fold less potent against DR/5-HTR than its analog fluphenazine (FLU).

**Results:**

CWHM-974 was more potent than FLU against a panel of cancer cell lines, thus clearly demonstrating that its anti-cancer effects were independent of DR/5-HTR signaling. Our results further suggested that calmodulin (CaM) binding may be necessary, but not sufficient, to explain the anti-cancer effects of CWHM-974. While both FLU and CWHM-974 induced apoptosis, they induced distinct effects on the cell cycle (G0/G1 and mitotic arrest respectively) suggesting that they may have differential effects on CaM-binding proteins involved in cell cycle regulation.

**Discussion:**

Altogether, our findings indicated that the anti-cancer efficacy of the CWHM-974 is separable from DR/5-HTR antagonism. Thus, reducing the toxicity associated with phenothiazines related to DR/5-HTR antagonism may improve the potential to repurpose this class of drugs to treat brain tumors and/or brain metastasis

## Introduction

Metastasis to the central nervous system (CNS) develops in nearly 30% of cancer patients, and the incidence is likely rising because of longer survival durations, better surveillance, increased detection and an aging population (1). Certain primary cancers have a higher propensity to develop brain metastasis such as lung cancer, breast cancer and melanoma which altogether account for 67-80% of patients with brain metastasis (2). Patients with lung cancer have the highest rates of brain metastasis at diagnosis while those with melanoma have the highest risk of presenting with brain metastasis during the course of their disease (3). Even with treatments involving surgical intervention, irradiation, and chemotherapy, brain metastasis is associated with a dismal prognosis as median survival is 4-10 months (lung); 10 months (breast); and 6 months (melanoma) and is often considered fatal (3). The blood-brain barrier (BBB) often limits the efficacy of classical chemotherapy as well as targeted therapies for patients with brain cancer metastasis due to poor drug penetration (1, 4, 5). This has driven many efforts to improve BBB penetration in modern small-molecule targeted therapeutics, but the efficacy of such targeted therapeutics has remained limited for lung, breast, and melanoma (1). Immunotherapy is a promising approach, but how the immuno-privileged state of the brain may affect this modality remains to be seen. Therefore, brain metastasis remains a significant unmet clinical need.

The challenges of developing new drugs motivated the consideration of alternatives such as “drug repurposing” or “drug repositioning”. Repurposing existing drugs from one therapeutic area to treat other diseases, has the significant advantage of shortening the time required for clinical application based on known pharmacology and toxicology (6). Since psychiatric drugs can effectively penetrate the BBB, they already possess a key attribute for an effective anti-brain metastasis therapy (7). To date, there has been very little clinical success with this approach using several major classes of psychiatric drugs (7). However, these clinical efforts have involved FDA-approved psychiatric drugs, and the most significant pharmacologic liability to the antipsychotic drugs scaffold is the dose-limiting toxicity related to dopamine receptor (DR)-dependent sedation and neuroleptic malignant syndrome (8).

The phenothiazines (PTZs), discovered by Erlich in the 1920’s and developed as anti-psychotic agents in the 1950’s, inhibit dopamine receptors (DRs), particularly the D2-type, as well as serotonin receptors (5-HTRs) (9). Motivated by epidemiologic studies indicating that the use of PTZs may be associated with reduced cancer risk, numerous efforts have investigated the potential anti-cancer properties of these drugs over the past couple of decades (10, 11). Most of these studies have focused on chlorpromazine (CPZ), thioridazine and trifluoperazine (10, 11). However, to date, only thioridazine completed a phase 1 clinical trial (NCT02096289) for relapsed or refractory acute myeloid leukemia with preliminary results suggesting that DRD2 represents a potential therapeutic target for this disease (12). CPZ is currently being investigated in a phase 1 clinical trial (NCT05190315) for newly diagnosed glioblastoma, but no results have been published to date. The latter concept originated from studies that CPZ inhibits cytochrome c oxidase subunit 4 isoform 1 (COX4-1) which is expressed at high levels in chemoresistant glioma cells (13).

Comparatively, few studies have involved fluphenazine (FLU) (10, 11). While these studies show that FLU is cytotoxic (IC_50_ 5-20 µM) to a variety of cancer cell types *in vitro* (11, 14), FLU has not been extensively evaluated in pre-clinical animal models of cancer. Numerous anti-cancer mechanisms-of-action have been ascribed to PTZs including, but not limited to, antiproliferative and pro-apoptotic effects that may stem from disruption of Ca^2+^-signaling through calmodulin (CaM) binding, inhibition of cytochrome c oxidase (CcO, complex IV) activity, disruptions of MAPK/ERK, AKT/PI3K, WNT pathways; increased autophagy, and inhibition of cancer stem cell behavior (10, 11, 13, 15). However, whether these effects are dependent on the DR antagonism activity of PTZ remains uncertain. Several studies have concluded that the DR antagonism of thioridazine, trifluoperazine and FLU is key to their anti-cancer effects (16–23). However, this conclusion is largely based on correlative findings (16, 19, 21). Although some of the anti-cancer effects of thioridazine, trifluoperazine and FLU were abolished when expression of DR was silenced (17, 18, 20, 22, 23), these studies did not account for a fundamental aspect of PTZ pharmacology. Specifically, the PTZs are nanomolar antagonists of DRs, yet their anti-cancer effects are only evident at concentrations in the micromolar range (10). Thus, while DR inhibition may contribute in some cases to the anti-cancer effects of PTZs, their broad-spectrum anti-cancer effects may be DR-independent as reported in two studies (15, 20).

Here, we used medicinal chemistry to modify the PTZ scaffold retaining the desirable property of BBB penetration while enhancing anti-cancer activity and reducing dose-limiting toxicity. This study takes advantage of novel PTZ derivatives developed to treat cryptococcal meningitis which, like brain metastasis, invades brain tissue and thereby is sheltered from drugs by the BBB. A recently published study has described a series of novel N-benzyl-linked FLU derivatives with both improved anti-fungal activity and reduced DR antagonism developed with the hypothesis that reducing DR/5-HTR antagonism will open the therapeutic window for repurposing of PTZs as anti-fungal agents (24).

These previous efforts have resulted in the identification of a lead compound, CWHM-974, which is 100-1000-fold less potent than its parent compound FLU (micromolar *vs*. nanomolar affinity) for most of the DRs/5-HTRs (24). For this study, we compared the anti-cancer effects of CWHM-974 and its analog FLU against a panel of human cancer cell lines. Our results indicate that CWHM-974 is more potent against cancer cell lines compared to FLU suggesting that the anti-cancer effect of CWHM-974 is independent of DR signaling but is dependent on binding to CaM. Interestingly, although both compounds induced apoptosis, FLU induced a G1/G0 cell cycle arrest while CWHM-974 induced a G2/M arrest. This discordance points toward different mechanisms of action, suggesting that effects shared by the members of the PTZs, such as CaM binding, are unlikely to be solely responsible for the anti-cancer effects of PTZs. Finally, we found that CPZ like CWHM-974, induces a G2/M arrest characterized by a late mitotic arrest. Although CWHM-975 lacks DR/5-HTR binding activity, it retains the BBB penetrating properties of PTZs and accumulates in brain tissue. While more study is needed to understand the mechanisms of action of CWHM-974, this new PTZ derivative demonstrates that DR/5-HTR antagonism does not underlie the broad-spectrum anti-cancer activity of PTZs.

## Materials and methods

### Cell culture

Cell lines: MDA-MB-231, ZR-75-1, HCC1806, U87, U251, DU145, 22Rv1, PC-3, LNCaP, HT-29, HCT-116, HepG2, A375, A101D, WM-266-4, Hs852T, RPMI7951, SKMEL28, A2058, RT4, TCCSUP, BxPC3, PANC1, 786-O, A498, MCF10A were obtained from the American Type Culture Collection (ATCC, Manassas, VA). R545 tet-inducible RasV12 melanoma cells (a kind gift from Dr. Lynda Chin, Harvard University) were cultured in RPMI1640 supplemented with 10% fetal bovine serum (FBS), 1% non-essential amino acids (NEAA) and 2µg/ml Doxycycline (Dox) to induce RasV12 expression (25). All cell lines were grown at 37°C in a 5% CO_2_ atmosphere in the ATCC-specified medium containing 10% FBS and 1% NEAA (**Table 1**).

**Table 1:** List of cell lines used for this study.

Tissue	Cell line	Media
Breast	MDA-MB-231	DMEM (10% FBS; 1% NEAA)
	MCF10A	
	HCC1806	RPMI1640 (10% FBS; 1% NEAA)
	ZR-75-1	
Prostate	PC-3	DMEM/F12 (10% FBS; 1% NEAA)
	LNCaP	RPMI1640 (10% FBS; 1% NEAA)
	DU145	
	22Rv1	
Large Intestine	HT29	DMEM (10% FBS; 1% NEAA)
	HCT116	
Pancreas	PANC1	DMEM (10% FBS; 1% NEAA)
	BxPC3	RPMI1640 (10% FBS; 1% NEAA)
Kidney	A498	DMEM (10% FBS; 1% NEAA)
	786-O	RPMI1640 (10% FBS; 1% NEAA)
Liver	HepG2	DMEM (10% FBS; 1% NEAA; L-Glut)
Urinary Tract	TCCSUP	RPMI1640 (10% FBS; 1% NEAA)
	RT4	McCoy’s 5 (10% FBS; 1% NEAA)
Central Nervous System	U87	DMEM/F12 (10% FBS; 1% NEAA)
	U251	
Skin	A375	DMEM (10% FBS; 1% NEAA)
	SKMEL28	
	RPMI16407951	
	Hs852T	
	A101D	
	WM-266-4	
	A2058	
Skin (mouse)	R545 tet-inducible RasV12	RPMI1640 (10% FBS ; 1% NEAA ; ± 2µg/ml Doxycycline)

### Pharmacologic reagents

W-7 hydrochloride (#0369, Tocris Bioscience, Bristol UK), chlorpromazine hydrochloride (CPZ) (C8138, Sigma, Saint-Louis, MO), fluphenazine hydrochloride (FLU) (PHR1792, Sigma, Saint-Louis, MO), CWHM-974 (24) and sulfoxide derivative of CWHM-974 (SLU-0010894) (26).

### Cell viability assay

Cells were seeded in 96-well plates at a suitable cell density for the growth rates of individual cell lines and the incubation time of the assay. After 24 h, cells were treated with a serial dilution series of FLU, W-7, CWHM-974, SLU-0010894 or chlorpromazine for 24h to 72h. Cell viability was determined using Cell-Titer-Blue assay (Promega) according to manufacturer’s instructions. The Synergy HT plate reader (Biotek, Winooski, VT) was used for signal quantification and the fluorescence values were normalized to the vehicle well for each cell line. Absolute IC_50_ and Growth rate inhibition GR_50_ (27) were calculated with GraphPadPrism software (GraphPad Software, Inc.).

### Cell cycle and apoptosis assay

A375 cells were seeded at 250,000 cells/well in 6-well plates and left to attach overnight. Cells were then treated with serial dilution series of FLU, W-7, CWHM-974 or CPZ for 24h. For cell cycle analysis, cells were detached with trypsin, fixed in ethanol 70%, treated with RNase A, mixed with propidium iodide, and processed with the Becton Dickinson LSR II flow cytometer. Analysis was performed with ModFit LT software. Apoptosis was assessed with the Annexin V Apoptosis Detection kit (Biolegend cat#640914, San Diego, CA) according to the manufacturer’s instructions. Samples were processed with the Becton Dickinson LSR II flow cytometer, and the analysis was performed with FlowJo software. Annexin V positive and PI negative cells were counted as apoptotic cells.

### Western blot

A375 cells were seeded at 250,000 cells/well in 6-well plates and left to attach overnight. Cells were then treated with serial dilution series of FLU, W-7, CWHM-974 or chlorpromazine for 24h. Treated cells were harvested and lysed with 100ul 1X lysis buffer containing β-mercaptoethanol (4X Laemmli Sample Buffer, Biorad). 25 to 50 µg of protein were separated on SDS-polyacrylamide gels and transferred on PVDF membranes (Immobilon-FL) before blocking (Intercept Blocking Buffer PBS, LI-COR) and incubation with primary antibody overnight at 4°C (**Table 2**). Membranes were then incubated with the corresponding secondary antibody for 1 h at room temperature. The membranes were scanned with an Odyssey infrared imaging system (LI-COR Biosciences, Lincoln NE), and expression of proteins was normalized to actin expression.

**Table 2:** List of antibodies used for this study.

Target	Source	Dilution
**β-Actin**	Clone AC-15, A1978, Sigma (Saint-Louis, MO)	1/10,000
**cdc25C**	Clone 5H9, CS#4688, Cell Signaling Technology (Danvers, MA)	1/1,000
**p-cdc25C (Ser216)**	Clone 63F9, CS#4901, Cell Signaling Technology (Danvers, MA)	1/1,000
**cdc2 (CDK1)**	CS#9112, Cell Signaling Technology (Danvers, MA)	1/1,000
**p-cdc2 (CDK1) (Tyr15)**	Clone 10A11, CS#4539, Cell Signaling Technology (Danvers, MA)	1/1,000
**Cleaved caspase3**	Clone 5A1E, CS#9664, Cell Signaling Technology (Danvers, MA)	1/1,000
**Pericentrin**	ab4448, Abcam (Cambridge, UK)	1/100
**Alpha-Tubulin**	12G10, DSHB (Iowa City, IA)	1/100
**Donkey anti-mouse IgG IRDye 800CW**	925-32212, LI-COR Biosciences, Lincoln NE	1/20,000
**Goat anti-rabbit IgG IRDye 680RD**	925-68071, LI-COR Biosciences, Lincoln NE	1/20,000
**Goat anti-Rabbit IgG Alexa Fluor™ 568**	cat#A21069, ThermoFisher Scientific, Waltham MA	1/500
**Goat anti-Mouse IgG Alexa Fluor™ 488**	cat#A11017, ThermoFisher Scientific, Waltham MA	1/500

### Immunofluorescence

Cells were seeded on glass coverslips and treated with 20µM FLU, CWHM-974 or chlorpromazine for 24h. At the end of the incubation, cells were fixed in 4% PFA, permeabilized in PBS-0.2% Triton X100, and blocked in PBS-2.5% BSA before antibody incubation. Cells were incubated with anti-Tubulin and anti-Pericentrin antibodies overnight at 4°C, and then with secondary antibodies for 1 h at room temperature (**Table 1**). All antibodies were diluted in PBS-2.5% BSA. The coverslips were then mounted on cover glass with a DAPI (4’,6-diamidino-2-phenylindole)-containing mounting media (Invitrogen P36962, Waltham, MA), and imaged with a Leica SP8 confocal microscope.

### Human calmodulin binding assay

Steady-state fluorescence emission spectra were collected as previously described (28). Briefly, emission spectra were collected in 1 nm increments from 290-350 nm at ambient temperature using a Fluorolog 3 spectrofluorometer with a xenon short arc lamp and an excitation of 277 nm. Recombinant human calmodulin was diluted to a working concentration of 50 µg/mL in assay buffer containing 50 mM HEPES pH 7.5, KCl 100 mM, and CaCl_2_ 10 mM. The compound was titrated as reported with a corresponding DMSO control for background subtraction. Data was collected using the FluorEssence™ software and analyzed using GraphPad Prism. Dose-response was considered from background and solvent-corrected data for the emission maximum of 314 nM.

### Pharmacological characterization of CWHM-974 in mice

Twenty-one female CD-1 mice were dosed IP with 10 mg/kg CWHM-974. Whole blood was collected in a syringe coated with ACD. Plasma was processed from whole blood by centrifugation at 10,000 rpm for 10 minutes. Brain tissue was harvested and gently washed with 1x PBS to remove residual circulating blood. The tissue was weighed, and snap-frozen in liquid nitrogen. Plasma Processing: For the standards and quality controls, 98 μl & 98.8 μl of blank vendor plasma was added to a microfuge tube and spiked with 2 & 1.2 μl of initial standard. Standards, quality controls & samples of 100 μL were then precipitated with 200 μL of methanol containing 0.15% formic acid and 12.5 ng/mL IS (final concentration). The samples were vortexed 15 sec, incubated at room temperature for 10 min, and centrifuged at 13,200 rpm twice in a standard microcentrifuge. The supernatant was then analyzed by LC-MS/MS. Vendor supplied plasma used in standards and QCs: Bioreclamation, LLC; lot #MSE284936; ACD anticoagulant. Brain Processing: Brain tissues were homogenized in 3X volume of PBS (X - weight of the tissue). For the standards and quality control samples, 98 μl & 98.8 μl of pooled blank brain was added to a microfuge tube and spiked with 2 µl or 1.2 μl of initial standard. Standards, quality control samples of 100 µl were then precipitated with 200 µl of methanol containing 0.15% formic acid and 12.5 ng/ml IS (final concentrations). The samples were vortexed 15 sec, incubated at room temp for 10 min, and centrifuged at 13,200 rpm twice in a standard microcentrifuge. The supernatant was then analyzed by LC-MS/MS.

### Whole animal toxicity of CWHM-974

For escalating dose studies, CD-1 females (Envigo), 25-30 grams were given 0, 25, 50 or 100 mg/kg CWHM-974 via intraperitoneal (I.P) injection once daily for 6 consecutive days (n=3 mice per group). CWHM-974 was formulated with 5% DMSO in 20% Kolliphor RH 40 in D5W. Mice were weighed daily, and monitored for other symptoms of toxicity.

### Statistical analysis

Statistical analysis was conducted using one-way or two-way ANOVA followed by Bonferroni test, or Spearman correlation by GraphPad Prism as specified in figure legends (GraphPad, La Jolla, CA, USA). A *p* value < 0.05 was considered significant (* p<0.05, ** p<0.01, *** p<0.001, **** p<0.0001).

## Results

### CWHM-974 is more potent than FLU against a panel of cancer cell lines

**Figure 1:**
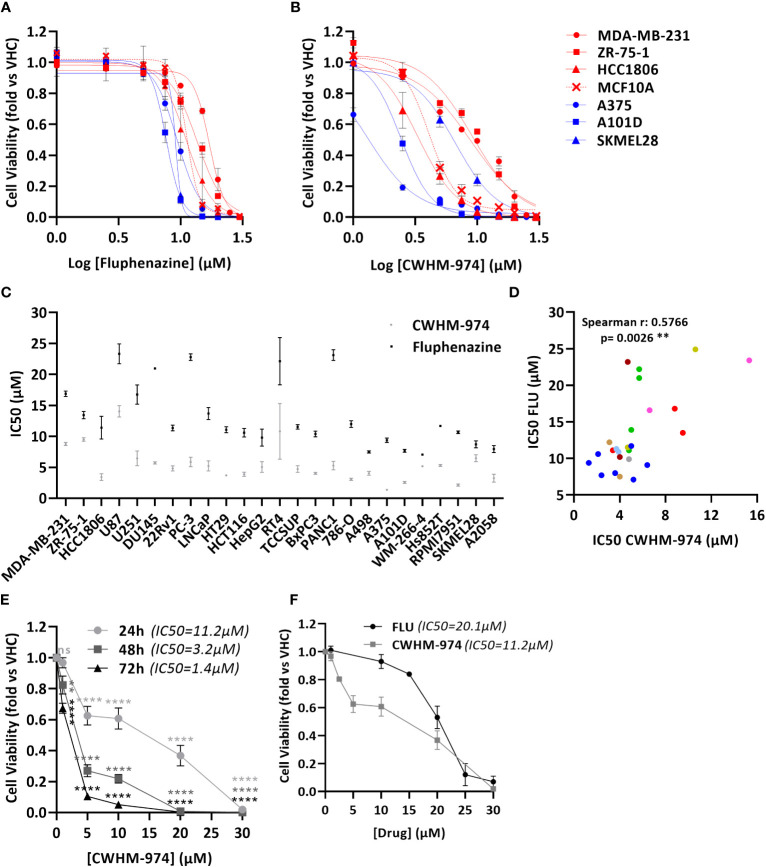
CWHM-974 is more potent than FLU against a panel of cancer cell lines. ***(*A, B*)*** Cells were treated for 72 h with serial dilution of FLU **(A)** or CWHM-974 **(B)**. Relative cell viability was plotted against the logarithm of drug concentration. **(C)** Absolute IC_50_ (72h) for FLU and CWHM-974 were determined using GraphPad Prism 6. **(D)** Correlation between IC_50_ (72h) for FLU and CWHM-974 (Spearman test). **(E)** Cell viability of A375 cells measured after 24h, 48h or 72 h treatment with serial dilution of CWHM-974. Two-way ANOVA followed by a Bonferroni test was used to assess the statistical significance of the dose effect. **(F)** Cell viability of A375 cells measured after 24h treatment with serial dilution of FLU or CWHM-974. **(A–F)** The results represent the means ± SEM of at least 3 independent experiments. ** p<0.01; **** p<0.0001; ns, not significant.

To definitively evaluate the role of DR/5-HTR antagonism of PTZs in the broad-spectrum anti-cancer activity, we took advantage of a compound recently developed from a PTZ scaffold, CWHM-974, that is 100-1000 less potent against DR/5-HTR while maintaining its antifungal potency (24). The anti-cancer effect of CWHM-974 was tested against a panel of cancer cell lines from different tissues of origin and compared to the anti-cancer effect of its analog FLU. Cell viability was measured after 72h of drug exposure, and absolute IC_50_ was calculated. Absolute IC_50_’s ranged between 1.37-14.03µM for CWHM-974 and 7.04-23.33µM for FLU (**Figures 1A-C**; **Supplementary Figures S1A, B**; **Table 3**). Interestingly, CWHM-974 was equally or more potent than FLU against all the cell lines tested (**Figure 1C**). However, while potency is correlated between these agents (**Figure 1D**), the increased potency of CWHM-974 over FLU is not strictly proportional and varies from a 1- to 7-fold increase. We also investigated whether cellular transformation influences the potency of both CWHM-974 and FLU. To do so, we assessed the sensitivity to both drugs of the R545 doxycycline inducible RasV12 murine melanoma cells (25) grown in the presence or absence of doxycycline. While CWHM-974 remained more potent than FLU, the transformation did not affect the sensitivity to these compounds (**Supplementary Figures S1C, D**). Additionally, the immortalized, but non-tumorigenic human breast epithelial MCF10A cells were amongst the most sensitive cells to both CWHM-974 and FLU (IC_50_ 4.3µM and 11.3 µM respectively) (**Figures 1A, B**).

**Table 3:** Calculated IC50 (µM) for fluphenazine, CWHM-974 and W-7 at 72h.

	Fluphenazine	CWHM-974	W-7
**R545 Tet-inducible RasV12 Dox (**–)	13.2	7.2	/
**R545 Tet-inducible RasV12 Dox(+)**	12.7	7.1	/
**MDA-MB-231**	16.9	8.8	34.6
**MCF-10A**	11.3	4.3	/
**HCC1806**	11.4	3.4	26.9
**ZR-75-1**	13.4	9.5	25.2
**PC-3**	22.8	5.9	40.8
**LNCaP**	13.7	5.2	36.6
**DU145**	21.0	5.7	37.3
**22Rv1**	11.3	4.8	39.1
**HT29**	11.1	3.7	26.4
**HCT116**	10.6	3.9	27.9
**PANC1**	23.1	5.3	32.7
**BxPC3**	10.4	4.0	38.1
**A498**	7.5	4.0	28.0
**786-O**	12.0	3.1	33.5
**HepG2**	9.8	5.0	26.8
**TCCSUP**	11.5	4.7	25.9
**RT4**	22.1	10.8	45.2
**U87**	23.3	14.0	56.8
**U251**	16.8	6.4	30.0
**A375**	9.4	1.4	21.8
**SKMEL28**	8.7	6.5	29.3
**RPMI7951**	10.7	2.1	26.5
**Hs852T**	11.7	5.3	24.5
**A101D**	7.7	2.6	26.9
**WM-266-4**	7.0	5.2	28.2
**A2058**	7.9	3.2	24.3

"/" means not applicable.

To examine whether CWHM-974- and FLU-induced toxicity is correlated with cell growth rate, we evaluated the sensitivity of the cell lines to these drugs by measuring the growth rate inhibition 50 (GR_50_), which accounts for differences in growth rates (27). We noted that across this panel of cell lines, the sensitivity to CWHM-974 and FLU as measured by IC_50_ and GR_50_ were well correlated (**Supplementary Figures S1E-F**). Finally, except for *HTR3A*, sensitivity of cancer cell lines to CWHM-974 and FLU did not correlate with mRNA expression of dopamine and serotonin receptors suggesting that these effects are independent of dopamine or serotonin receptor activity (**Supplementary Figure S1G**). Although *HTR3A* expression was positively correlated with sensitivity to CWHM-974, its expression is low to negligible (eg. no detectable mRNA expression in 12/25 cell lines) (**Supplementary Figure S1H**). Moreover, CHWM-974 does not exhibit detectable binding to 5-HT_3_ (24). Melanoma is amongst the most common cancers that metastasize to the brain, presenting a significant clinical challenge. We selected A375 cells, a melanoma cell line that displayed the highest sensitivity to CWHM-974 (IC_50_ 1.37µM) and high sensitivity to FLU (IC_50_ 9.35µM), to explore the toxicity mechanisms of both drugs. While the effect of CWHM-974 is evident within the first 24h, most of CWHM-974-induced toxicity takes place between 48h and 72h exposure (**Figure 1E**). We selected 24h as a time point for the evaluation of the toxicity mechanisms because, at that time point, CWHM-974 significantly decreased cell viability at a concentration of 5µM or higher (**Figure 1F**).

### FLU and CWHM-974 induce distinct cell cycle effects in A375 cells

**Figure 2:**
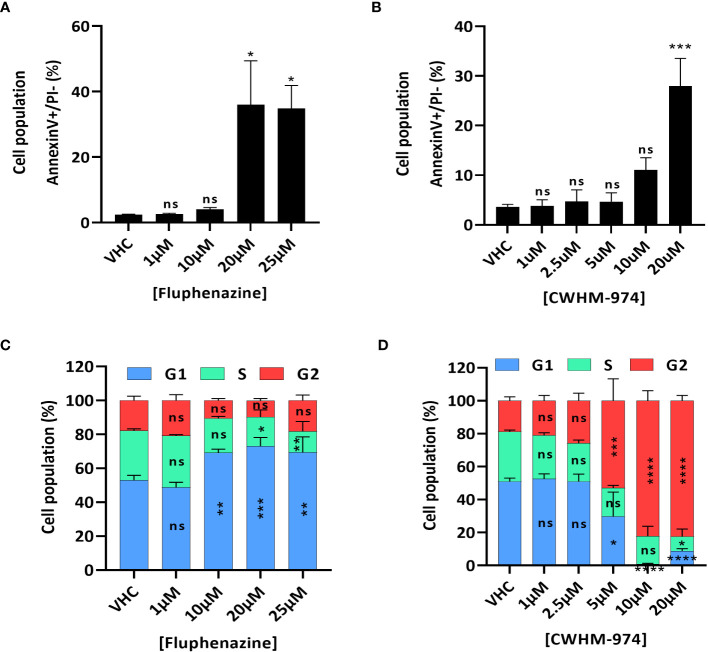
FLU and CWHM-974 induce distinct effects on cell cycle in A375 cells. **(A, D)** A375 cells were treated with FLU **(A, C)** or CWHM-974 **(B, D)** for 24h. Treated cells were harvested, and apoptosis induction was measured with an Annexin V staining **(A, B)**, and cell cycle progression was measured with PI staining **(C, D)**. One-way **(A, B)** or two-way **(C, D)** ANOVA followed by a Bonferroni test was used to assess the statistical significance of the dose effect. The results represent the means ± SEM of at least 3 independent experiments. * p<0.05; ** p<0.01; *** p<0.001; **** p<0.0001; ns, not significant.

To further examine the nature of the anti-cancer effect of FLU and CWHM-974, we investigated the type of cell death induced by FLU and CWHM-974 after 24h exposure. Because it was previously reported that FLU induces apoptosis (29, 30), we first evaluated the effect of FLU and CWHM-974 on apoptosis induction. As expected, both FLU (**Figure 2A**; **Supplementary Figure S2A**) and CWHM-974 (**Figure 2B**; **Supplementary Figure S2B**) induced apoptosis in A375 cells at concentrations ≥20µM as measured by an annexin V staining assay. CWHM-974 20µM also induced Caspase 3 cleavage in A375 cells confirming apoptosis as a mechanism of cell death (**Supplementary Figures S2C, D**). Since cell cycle arrest in G0/G1 is also a hallmark of the anti-cancer effect of FLU in a breast cancer cell line (29), we examined the effect of FLU and CWHM-974 on cell cycle regulation in A375 cells. Interestingly, while we confirmed that FLU induced a G0/G1 cell cycle arrest at concentrations ≥10µM (**Figure 2C**; **Supplementary Figure S2E**), CWHM-974 induced a G2/M arrest at concentrations ≥5µM (**Figure 2D**; **Supplementary Figure S2F**). the G2/M arrest induced by CWHM-974 was consistent with a decrease of cdc25c expression and CDK1 phosphorylation at concentrations ≥2.5µM (**Supplementary Figures S2G, H, I**).

### CWHM-974 and CPZ induce a late mitotic arrest

**Figure 3:**
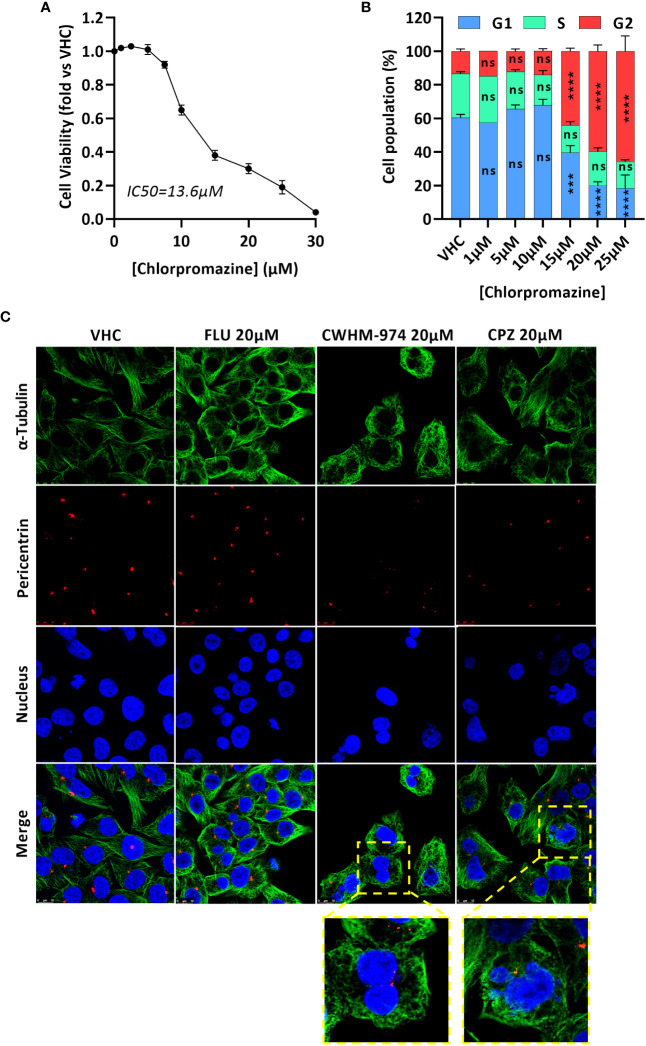
CWHM-974 and chlorpromazine induce a late mitotic arrest. **(A)** Cell viability of A375 cells was measured after 24h treatment with serial dilution of chlorpromazine. **(B)** Cell cycle progression (PI staining) was measured on A375 cells treated with chlorpromazine for 24h. Two-way ANOVA followed by a Bonferroni test was used to assess the statistical significance of the dose effect. **(C)** Cells were treated with DMSO, FLU (20µM), CWHM-974 (20µM) and chlorpromazine (20µM) for 24h. Cells were stained for α-tubulin (green), pericentrin (red) and nucleus (blue), and images were acquired using a Leica SP8 confocal microscope (x63). **(A, B)** The results represent the means ± SEM of 3 independent experiments. *** p<0.001; **** p<0.0001; ns, not significant.

While a G1/G0 cell cycle arrest was described for trifluoperazine (31–34), thioridazine (20, 35) and FLU (29), only CPZ is reported to induce a G2/M arrest, and more specifically to induce a mitotic catastrophe (36–38). We first confirmed that CPZ-induced toxicity in A375 cells correlated with a cell cycle arrest in G2/M (**Figures 3A, B**). We then asked if the arrest induced by CWHM-974 and CPZ occurred in the G2 or M phase and occurred via similar mechanism between these two agents. To answer this question, we performed immunofluorescence staining for α-tubulin (microtubules), pericentrin (centrioles) and DNA (nucleus) on cells treated for 24h with 20µM FLU, CWHM-974 and CPZ (**Figure 3C**). When cells were treated with DMSO, all phases of the cell cycle were represented, including all the different features of the mitotic phase. When cells were treated with FLU, we observed a dramatic decrease in the number of cells in mitosis consistent with the accumulation of cells in G0/G1. However, when cells were treated with CPZ and CWHM-974, cells often appeared binucleated consistent with a mitotic catastrophe. This phenotype was more severe in CWHM-974-treated cells. When cells appeared mononucleated, two centrioles were often present suggesting that the blockage occurred after cells entered mitosis. These observations were consistent with the results of the cell cycle analysis showing that at 20µM, only 69.8% of the cells treated with CPZ were in the G2/M phase compared to 82.4% of the cells treated with CWHM-974. These results suggest that, although the effect of CWHM-974 is more profound than the effect of CPZ, the mechanisms of action of these two drugs are similar.

### CaM binding is necessary but not sufficient for the anti-cancer effects of CWHM-974

**Figure 4:**
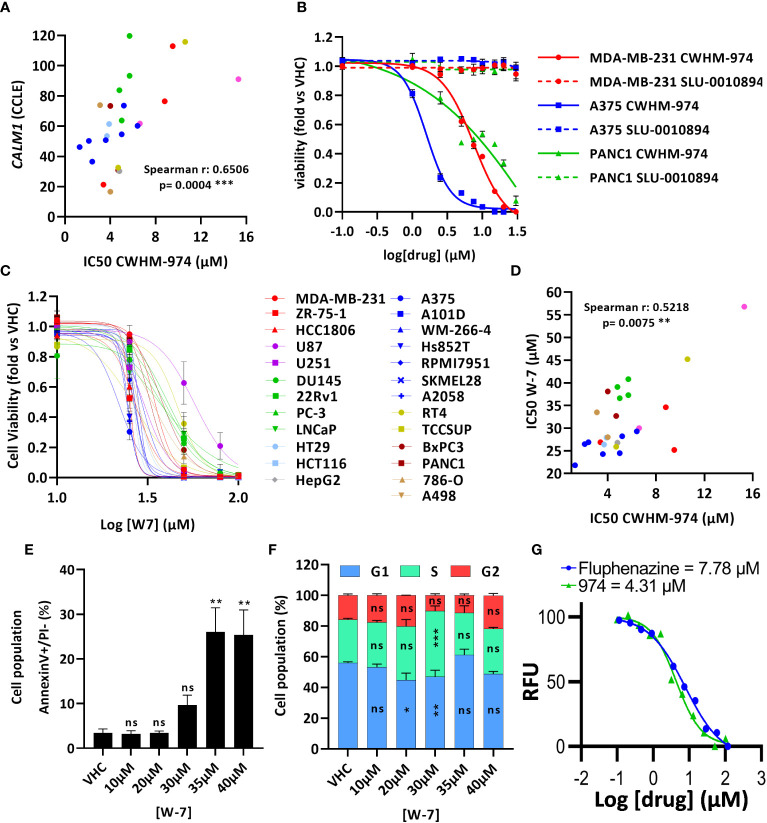
Calmodulin binding is necessary but not sufficient for the anti-cancer effects of CWHM-974. **(A)** Correlation between IC_50_ (72h) for CWHM-974 and expression of *CALM1* exported from the CCLE (Spearman test). **(B, C)** Cells were treated for 72h with serial dilution of SLU-0010894 **(B)** or W-7 **(C)**. Relative cell viability was plotted against the logarithm of drug concentration. **(D)** Correlation between IC_50_ (72h) for W-7 and CWHM-974 (Spearman test). **(E, F)** A375 cells were treated with W-7 for 24h. Treated cells were harvested, and apoptosis induction was measured with an Annexin V staining **(E)**, and cell cycle progression was measured with PI staining **(F)**. One-way **(E)** or two-way **(F)** ANOVA followed by a Bonferroni test was used to assess the statistical significance of the dose effect. **(G)** Calmodulin binding assay of CWHM-974 and FLU measuring intrinsic protein fluorescence at an emission of 314 nm from an excitation of 277 nm. Dose-response was fit using a non-linear regression model from GraphPad Prism. The experiment shown is representative of three independent replicates. **(A–G)** The results represent the means ± SEM of at least 3 independent experiments. * p<0.05; ** p<0.01; *** p<0.001; ns, not significant.

Montoya et al. have previously shown that the antifungal activity of the PTZ scaffold correlates with CaM antagonism, and that CWHM-974 blocks CaM-dependent nuclear localization of the transcription factor Crz1 (24). Therefore, we examined the role of CaM in the anti-cancer effects of CWHM-974. Using the Cancer Cell Line Encyclopedia (CCLE), we assessed the correlation between the expression of the three genes encoding human CaM (*CALM1*, *CALM2* and *CALM3*) and the IC_50_ values for CWHM-974 and FLU in the cancer cell lines tested. Interestingly, while all three genes encode the same protein, only the expression of *CALM1* significantly correlated with the sensitivity to CWHM-974 and FLU (**Figure 4A**; **Supplementary Figures S3A, D**). The sulfur atom of the PTZ ring is susceptible to metabolic oxidation *in vivo*, and the corresponding sulfoxide has been shown to decrease affinity for CaM (39). Consistent with that observation, Montoya et al. demonstrated that the sulfoxide derivative of thioridazine and CWHM-974 had no antifungal activity further supporting the correlation between anti-CaM activity and anticryptococcal activity (24, 26). We compared the effect of the sulfoxide derivative of CWHM-974 (SLU-0010894) to the effect of CWHM-974 on three cancer cell lines: A375 (Melanoma), MDA-MB-231 (breast) and PANC1 (pancreas) cell lines. As predicted, the sulfoxide derivative had no activity against all three cell lines tested (**Figure 4B**). We then evaluated the effect of W-7, a CaM inhibitor, on our panel of cancer cell lines (**Figure 4C**; **Table 3**). We also noted that sensitivity to W-7 measured after 72h exposure (IC_50_ 21.8-56.8µM), significantly correlated with the sensitivity to CWHM-974 and FLU (**Figure 4D**; **Supplementary Figure S3B**). While these results implicate CaM in the anti-cancer effects of CWHM-974, the cell death mechanisms triggered by CWHM-974 and W-7 appeared different, suggesting that CaM binding/inhibition alone is not sufficient to explain the anti-cancer properties of CWHM-974. Indeed, although both W-7- and CWHM-974-induced toxicity correlated with induction of apoptosis in A375 cells (**Figure 4E**; **Supplementary Figures S3C, E**), W-7 did not induce a cell cycle arrest as we observed for CWHM-974 in A375 cells (**Figure 4F**; **Supplementary Figure S3F**). Finally, by monitoring the intrinsic fluorescence of aromatic amino acids in CaM, we were able to demonstrate that CWHM-974 binds to mammalian CaM. (**Figure 4G**). Altogether these data suggest that CaM binding may be necessary but is not sufficient for the anti-cancer effects of CWHM-974.

### *In vivo* pharmacology of CWHM-974

**Figure 5:**
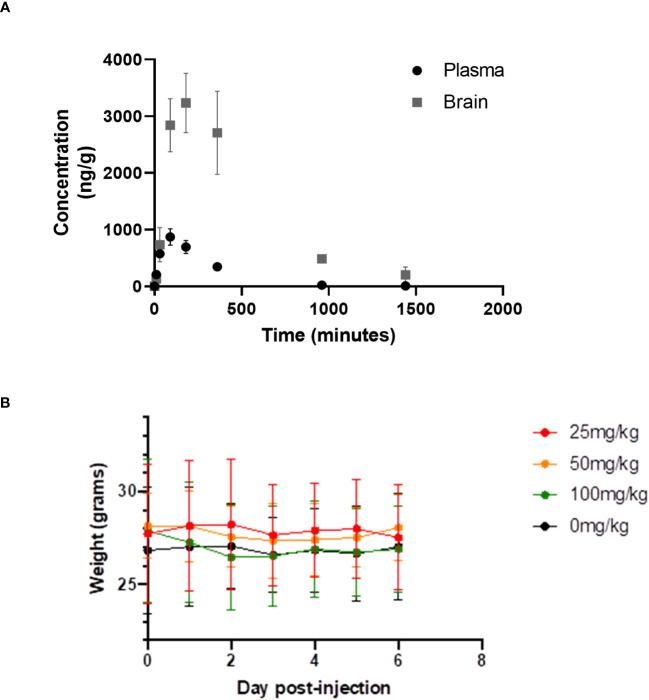
Pharmacology and toxicity of CHWM-974. **(A)** The plasma and brain concentrations of CHWM-974 over time after a 10mg/kg dose (I.P. injection) were determined as described in materials and methods. The points represent the mean concentration for three animals at each time point. **(B)** Dose escalation study for three single doses (I.P. injection) given to three mice per group. Weights for each mouse were determined over six days. The mean and standard deviations are shown.

We have measured the plasma and brain pharmacokinetics (PK) of CWHM-974. Based on prior studies of FLU, we evaluated plasma and brain CWHM-974 concentrations following a single bolus i.p. injection at 10mg/kg in 5% dextrose containing 5% DMSO and 20% Kolliphor RH40 (**Figure 5A**). We recorded C_MAX_ values of 0.8 µg/mL (2 µM) and 3.3 µg/mL (6 µM) for plasma and brain respectively, which is similar to that reported for FLU (12), indicating that the modifications made to CWHM-974 have not substantially changed the PK of FLU. The LD_50_ of FLU in mice is 89 mg/kg. Dose escalation with CWHM-974 indicates that it can be dosed up to 90 mg/kg with no weight loss or overt toxicity including sedation over the preceding six days (**Figure 5B**).

## Discussion

Repurposing psychiatric drugs such as PTZs is one approach that has been considered to treat primary brain tumors and brain metastasis. While broad-spectrum anti-cancer effects of PTZs have been reported previously, their mechanisms-of-action remain elusive. Some studies have shown DR-dependent effects of PTZ’s in certain cancer cell lines (16–19, 21–23), while other studies have demonstrated DR-independent effects (15, 20). Moreover, PTZs are nanomolar antagonists of DRs/5HTRs, yet their broad-spectrum anti-cancer effects are only evident at micromolar concentrations. Thus, the role of DRs/5-HTRs in the anti-cancer effects of PTZs is context dependent and may be dispensable. This issue is important because to achieve high concentrations in the brain, it will be necessary to avoid the dose-limiting toxicity associated with DR-dependent sedation and neuroleptic malignant syndrome. Previously, Montoya et al. described a novel PTZ derivative, CWHM-974, which is 100-1000-fold less potent than its parent compound FLU (micromolar *vs*. nanomolar affinity) for most of the DRs/5-HTRs to develop anti-fungal agents (24). Here we have explored the anti-cancer effects of CWHM-974.

When tested against a broad panel of cancer cell lines, CWHM-974 was, overall, more potent than its analog FLU. This was consistent with the results of Montoya et al. showing that CWHM-974 was also more potent as an antifungal agent (24). Our data are also consistent with the literature indicating that the anti-cancer effects of CWHM-974 occur in the micromolar concentration range (14). Cellular transformation did not seem to be a determining factor in the sensitivity to these compounds as the doxycycline-inducible expression of RasG12V in the R545 murine melanoma cell line had no effect on sensitivity to CWHM-974 and FLU. This raises a question whether CWHM-974 will be selectively cytotoxic to cancer cells *vs* normal cells. However, we have yet to test the effect of these compounds on primary human cells. Moreover, we show that when administered to mice at doses achieving micromolar exposure level *in vivo*, overt toxicity is not observed. Importantly our results establish that the broad spectrum anti-cancer effect of CWHM-974 is independent of DR and 5-HT receptors, since CWHM-974 demonstrates greater anti-cancer potency than FLU, but is a 100-1000-fold less potent inhibitor of DR/5-HTRs. Moreover, there is no correlation between the anti-cancer potency of CWHM-974/FLU and the mRNA expression of DR/5-HTRs across the panel of cell lines that we evaluated.

As melanoma not only commonly metastasizes to the brain but also was amongst the most sensitive cell lines tested in our study we chose the human melanoma cell line A375 (CWHM-974 IC_50_ 1.37µM), to study the mechanisms of action of CWHM-974 and FLU. Both compounds induced apoptosis at concentrations ≥20µM which is consistent with previous studies describing induction of apoptosis with FLU (29, 30). Surprisingly, FLU and CWHM-974 had very distinct effects on cell cycle regulation with FLU inducing a G0/G1 cell cycle arrest at concentrations ≥10µM, and CWHM-974 inducing a G2/M arrest at concentrations ≥5µM. Consistently, FLU along with two additional PTZs, trifluoperazine and thioridazine, have previously been reported to induce a cell cycle arrest in G0/G1 in different cancer models such as TBNC, lymphoma and melanoma (20, 29–35). Mechanistically, the arrest in G0/G1 was associated with the inactivation of PI3K/AKT and JNK/MAPK signaling pathways and a disruption of the cyclin-CDK complexes (29–35). However, other PTZs, such as CPZ and triflupromazine, were reported to induce a G2/M arrest, and more particularly a mitotic arrest in glioma, colorectal, lung and oral cancers (36–38, 40–42). CPZ and triflupromazine were for example described as inhibitors of KSP/Eg5 activity leading to the accumulation of cells with monopolar spindle and a mitotic arrest (36) (42). We confirmed that, similar to CWHM-974, CPZ induces a cell cycle arrest in G2/M in A375 cells and that, particularly, both drugs induce a late mitotic arrest characterized by the accumulation of multinucleated cells.

Numerous anti-cancer mechanisms of action have been ascribed to PTZs. The CaM-inhibiting activity of PTZ was one of the first mechanisms to be studied (39, 43–45), providing support for the repurposing of this class of drugs in cancer. Several studies have demonstrated novel mechanisms-of-action of these drugs in cancer cells including, but not limited to, inhibition of cytochrome c oxidase activity, disruptions of MAPK/ERK, AKT/PI3K, WNT pathways; increased autophagy, and inhibition of cancer stem cell behavior (10, 11, 13). In 2017, Montoya et al. demonstrated that the antifungal activity of PTZs, including CWHM-974, was dependent on CaM expression and activity (24). Consistent with this, recent work performed in glioblastoma multiforme suggesting that the cytotoxicity of DR antagonists involves calcium signaling mechanisms and not DR antagonism (15). Our results indicate that CaM binding is necessary for the anti-cancer effects of CWHM-974 because: 1) We demonstrate that CWHM-974 directly binds to CaM; 2) CALM1 expression significantly correlates with sensitivity to both FLU and CWHM-974; 3) the sulfoxide derivative of CWHM-974, which lacks CaM binding activity, had no anti-cancer effects; and 4) sensitivity to both FLU and CWHM-974 significantly correlates with sensitivity to the CaM inhibitor W-7. However, CaM binding alone is not sufficient to explain the anti-cancer effects of CWHM-974 because CWHM-974 and its analog FLU which both bind to CaM yield divergent effects on the cell cycle in A375 cells. CaM is a multifunctional calcium-binding protein which, by interacting with many other proteins, mediates a diverse range of calcium-sensitive cellular functions and signaling pathways that contribute to tumorigenesis (46). The role of CaM in cell cycle regulation has been extensively studied and, depending on the CaM-binding protein partner engaged, CaM can exert control at different stages of the cell cycle (46). For example, CaM was shown to regulate the G1/S transition through its association with Cdk4/cyclin D1 (47), cyclin E1 (48, 49) or CaMK-I (50, 51). On the other hand, CaM can control the G2/M transition via CaMK-II (47, 52) or Polo-like kinase 1 (Plk1) (53). Finally, CaM was shown to play critical roles during mitosis and cytokinesis. It was demonstrated that active CaM is mostly localized to the nucleus in preparation for mitosis and associated with mitotic spindles and centrosomes during mitosis (54, 55). A more recent study also established that CALM1-3 knockout induces an arrest in M phase characterized by a multinucleated senescent state (56). In addition, CaM binds and regulates some proteins involved in mitosis regulation such as MLCK, Aurora B (57, 58) and Aurora-A kinase (AURKA) (59). Overall, this emphasizes the occurrence of multiple and divergent CaM-regulated control mechanisms acting at the different phases of the cell cycle, which may explain how different PTZ compounds that bind to CaM could have different effects on cell cycle regulation.

In this study, we demonstrated that a novel PTZ derivative, CWHM-974, was more potent than FLU against a panel of cancer cell lines. More importantly, our data indicate that the anti-cancer effects of CWHM-974, while independent of dopamine and serotonin receptor signaling, were dependent on calmodulin binding. Interestingly, our data highlight that the anti-cancer effects of different PTZs is associated with distinct effects on cell cycle regulation. While this could be related to the alteration of CaM interaction with different binding partners that control different phases of the cell cycle, we cannot exclude that the anti-cancer effects of PTZs and their effect on cell cycle regulation might be mediated by distinct mechanisms that might be, in some cases, CaM-independent. CHWM-974, like its parent compound FLU, exhibits robust uptake in the brain reaching levels of 6µM in a single dose, without eliciting acute toxicity. This finding is encouraging for the anti-cancer potential of CWHM-974 for 2 reasons: First, as we show, the IC_50_ values for cancer cell line toxicity range from 1-15 µM across a broad spectrum of cancer cell lines are within the range of the brain C_MAX_ value. Second, the brain C_MAX_ value is ~15-fold lower than many of the DRs and serotonin receptors tested. Taken together, our data support further evaluation of CWHM-974 in animal models of primary brain cancer and brain metastasis.

## Data availability statement

The original contributions presented in the study are included in the article/**Supplementary Material**. Further inquiries can be directed to the corresponding author.

## Ethics statement

Ethical approval was not required for the studies on humans in accordance with the local legislation and institutional requirements because only commercially available established cell lines were used. The animal study was approved by Institutional Animal Care and Use Committee (IACUC), University of Iowa. The study was conducted in accordance with the local legislation and institutional requirements.

## Author contributions

MV: Conceptualization, Data curation, Formal Analysis, Investigation, Methodology, Validation, Writing – original draft, Writing – review & editing. AV: Investigation, Methodology, Writing – review & editing. SG: Data curation, Investigation, Methodology, Writing – review & editing. AF: Writing – review & editing, Data curation, Investigation, Methodology. AJ: Data curation, Investigation, Methodology, Writing – review & editing. SB: Data curation, Investigation, Methodology, Writing – review & editing. DK: Conceptualization, Data curation, Funding acquisition, Investigation, Methodology, Project administration, Resources, Supervision, Writing – review & editing. MM: Conceptualization, Data curation, Funding acquisition, Investigation, Methodology, Project administration, Resources, Supervision, Writing – review & editing. MH: Conceptualization, Funding acquisition, Investigation, Methodology, Project administration, Resources, Supervision, Writing – review & editing.

## Glossary

**Table:**

5-HTR	Serotonin receptor
AKT	Protein kinase B
AURKA	Aurora-A kinase
BBB	Blood-brain barrier
BME	β-mercaptoethanol
CaM	Calmodulin
CaMK	Ca^2+^/calmodulin-dependent protein kinase
CCLE	Cancer cell line encyclopedia
CDK	Cyclin dependent kinase
CNS	Central nervous system
COX4-1	Cytochrome c oxidase subunit 4 isoform 1
CPZ	Chlorpromazine
DMSO	Dimethyl sulfoxide
Dox	Doxycycline
DR	Dopamine receptor
ERK	Extracellular signal-regulated kinase
FBS	Fetal bovine serum
FDA	Food and Drug Administration
FLU	Fluphenazine
GR_50_	Growth rate inhibition 50
IC_50_	Half maximal inhibitory concentration
JNK	Jun N-terminal kinase
KSP/Eg5	Kinesin Spindle Protein Eg5
LC-MS/MS	Liquid chromatography-tandem mass spectrometry
LD_50_	Lethal dose 50
MAPK	Mitogen-activated protein kinase
MLCK	Myosin Light Chain Kinase
NEAA	Non-essential amino acid
PBS	Phosphate-buffered saline
PFA	Paraformaldehyde
PI	Propidium iodide
PI3K	Phosphoinositide 3-kinase
Plk1	Polo-like kinase 1
PTZ	Phenothiazine
PVDF	Polyvinylidene difluoride
QC	Quality Control
Ras	Rat sarcoma virus
SDS	Sodium dodecyl sulfate
WNT	Wingless-related integration site
