# Supplementary material for: The anti-cancer efficacy of a novel phenothiazine derivative is independent of dopamine and serotonin receptor inhibition
Source: Front Oncol. 2023 Oct 16;13:1295185. doi: 10.3389/fonc.2023.1295185 (PMC10613967; doi:10.3389/fonc.2023.1295185)

Supplementary Figures

Supplementary Fig. S1.
(A,B) Cells were treated for 72 h with serial dilution of FLU (A) or CWHM-974 (B). Relative cell viability was plotted against the logarithm of drug concentration. **(C)** Absolute IC_{50} (72h) for FLU and CWHM-974 was calculated for R545 Tet-inducible RasG12V melanoma cells in presence of absence of doxycycline 2µg/ml. One-way (ANOVA followed by a Bonferroni test was used to assess the statistical significance. **(D)** Confirmation of Ras^{G12V} induction in R545 Tet-inducible Ras^{G12V} melanoma cells by western blot. **(E,F)** Growth rate inhibition 50 (GR_{50}) was calculated and correlation between Absolute IC_{50} (72h) and GR_{50} (72h) was evaluated for FLU (E) and CWHM-974 (F) (Spearman test). **(G)** Correlation between the expression of dopamine and serotonin receptors (extracted from the CCLE) and the IC_{50} (72h) for FLU and CWHM-974 was calculated (Spearman test). **(H)** Correlation between *HTR3A* expression (extracted from the CCLE) and the IC_{50} (72h) for CWHM-974 (Spearman test). **(A-F)** The results represent the means ± SEM of at least 3 independent experiments.

Supplementary Fig. S2.
(A,B,E,F) Representative flow cytometry plots for the Annexin V staining (A,B) and cell cycle progression (E,F). **(C-D)** The level of cleaved caspase-3 was measured by western blot as a marker of apoptosis. (D) Quantification of 3 independent western blots. One-way ANOVA followed by a Bonferroni test was used to assess the statistical significance of the dose effect. **(G-I)** The levels of p-cdc25C, cdc25C, p-CDK1 and CDK1 was measured by western blot as a marker of cell cycle progression through G2/M. (H,I) Quantification of 3 independent western blots. Two-way ANOVA followed by a Bonferroni test was used to assess the statistical significance of the dose effect. **(C,G)** shows representative Western blots. **(D,H,I)** The results represent the means ± SEM of at least 3 independent experiments.

Supplementary Fig. S3.
(A) Correlation between IC_{50} (72h) for FLU and expression of *CALM1* exported from the CCLE (Spearman test). **(B)** Correlation between IC_{50} (72h) for W-7 and FLU (Spearman test). **(C)** Cell viability of A375 cells measured after 24h treatment with serial dilution of W-7. **(D)** Correlation between IC_{50} (72h) for FLU and CWHM-974 and the expression of *CALM1*, *CALM2* and *CALM3* exported from the CCLE (Spearman test). **(E,F)** Representative flow cytometry plots for the Annexin V staining displayed in Fig. 4E (E) and cell cycle progression displayed in Fig. 4F (F). **(A-C)** The results represent the means ± SEM of at least 3 independent experiments.

Supplementary Fig. S4.
Original blot for Fig. S1D.

Supplementary Fig. S5.
Original blot for Fig. S2C.

Supplementary Fig. S6.
Original blot for Fig. S2G.

Supplementary Fig. S1.

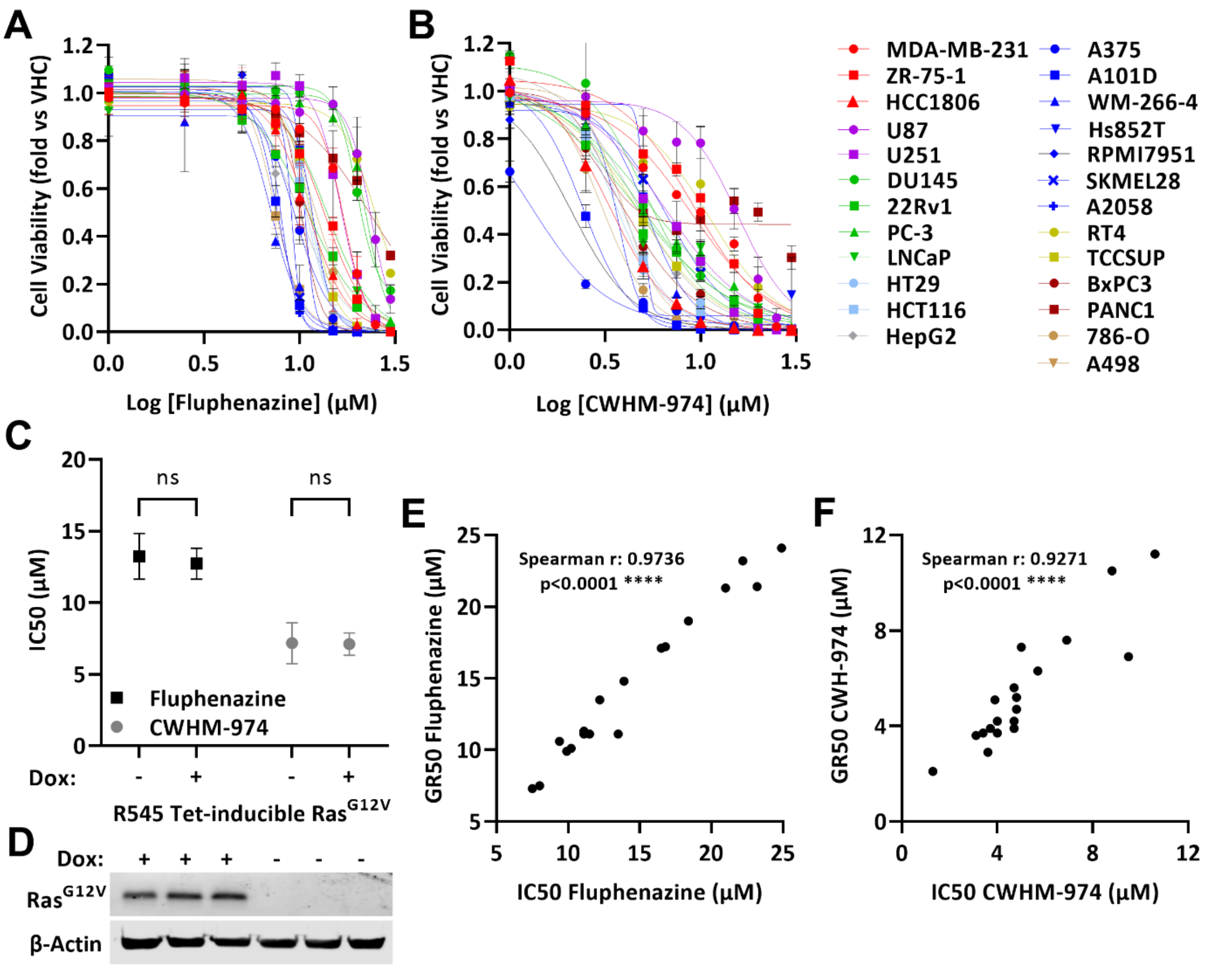

G

Spearman test (r , P value)	IC50 CWHM-974	IC50 Fluphenazine
DRD1	0.1002 ; 0.6335 ns	0.2673 ; 0.1964 ns
DRD2	-0.1572 ; 0.453 ns	0.06468 ; 0.7587 ns
DRD3	-0.1417 ; 0.4994 ns	-0.08494 ; 0.6865 ns
DRD4	-0.04158 ; 0.8436 ns	0.1019 ; 0.6278 ns
DRD5	-0.07175 ; 0.7332 ns	-0.06567 ; 0.7551 ns
HTR1A	0.1186 ; 0.5723 ns	0.125 ; 0.5515 ns
HTR1B	-0.3802 ; 0.0608 ns	0.06177 ; 0.7693 ns
HTR1D	-0.209 ; 0.3159 ns	0.09771 ; 0.6422 ns
HTR1E	0.2225 ; 0.2851 ns	-0.03269 ; 0.8767 ns
HTR1F	0.1291 ; 0.5386 ns	-0.1042 ; 0.62 ns
HTR2A	0.1611 ; 0.4418 ns	0.04439 ; 0.8331 ns
HTR2B	-0.1956 ; 0.3487 ns	-0.3067 ; 0.136 ns
HTR2C	0.0944 ; 0.6535 ns	-0.1606 ; 0.443 ns
HTR3A	**0.421 ; 0.0361 ***	0.09564 ; 0.6493 ns
HTR3B	0.08345 ; 0.6917 ns	-0.1133 ; 0.5896 ns
HTR3C	-0.2446 ; 0.2387 ns	0.0966 ; 0.646 ns
HTR3D	-0.2267 ; 0.2759 ns	-0.04247 ; 0.8403 ns
HTR3E	-0.0943 ; 0.6539 ns	-0.1793 ; 0.3912 ns
HTR4	-0.07999 ; 0.7039 ns	-0.1238 ; 0.5554 ns
HTR5A	-0.1431 ; 0.4949 ns	-0.0899 ; 0.6691 ns
HTR6	-0.08657 ; 0.6807 ns	-0.1047 ; 0.6186 ns
HTR7	-0.1598 ; 0.4455 ns	-0.1839 ; 0.3789 ns

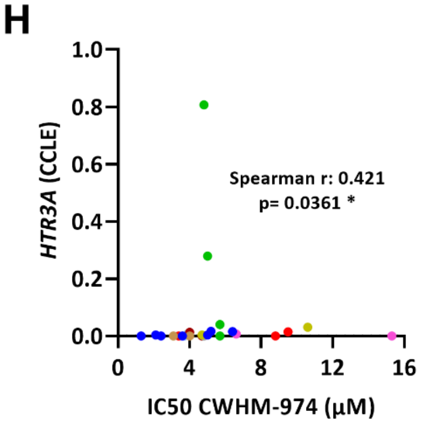

Supplementary Fig. S2.

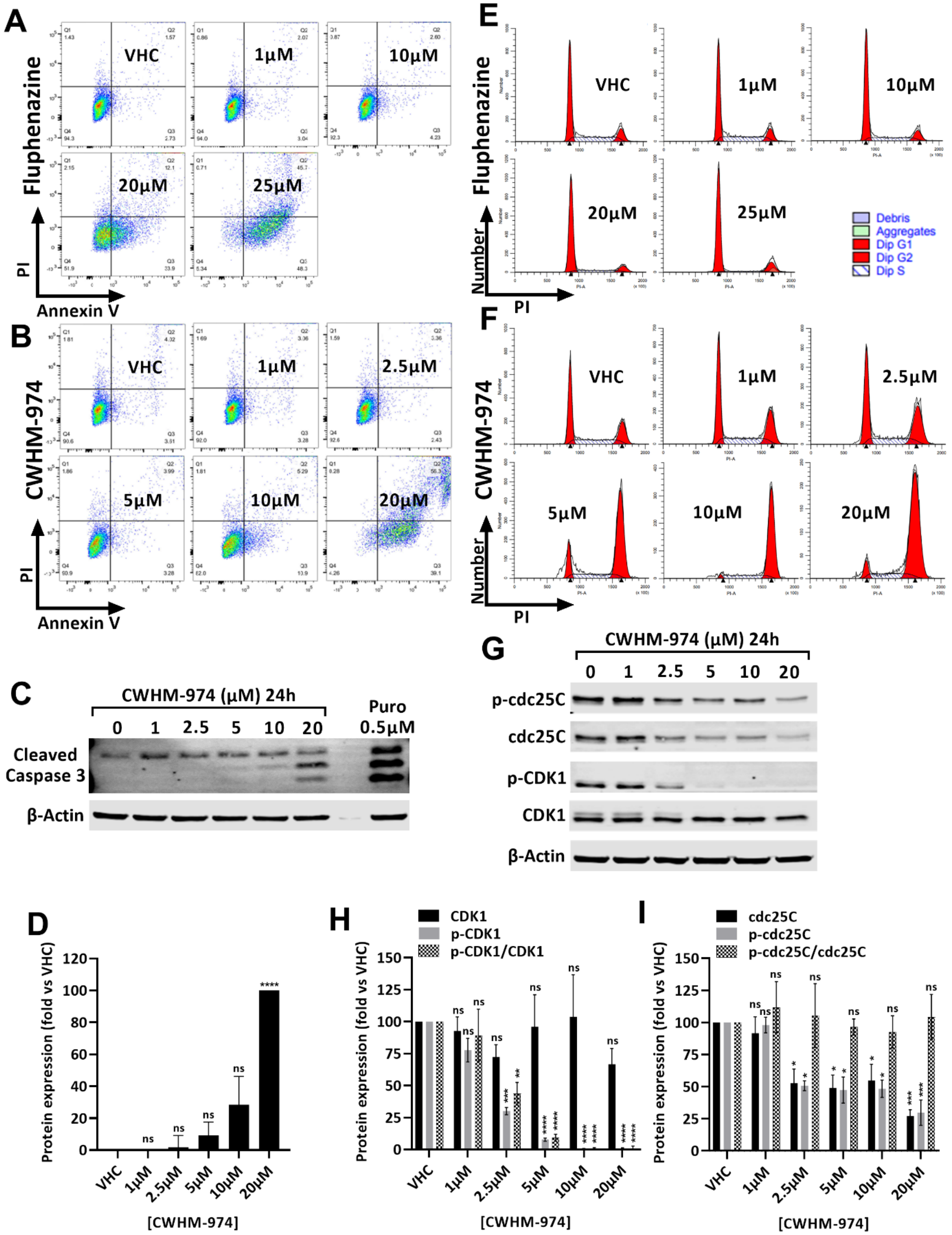

Supplementary Fig. S3.

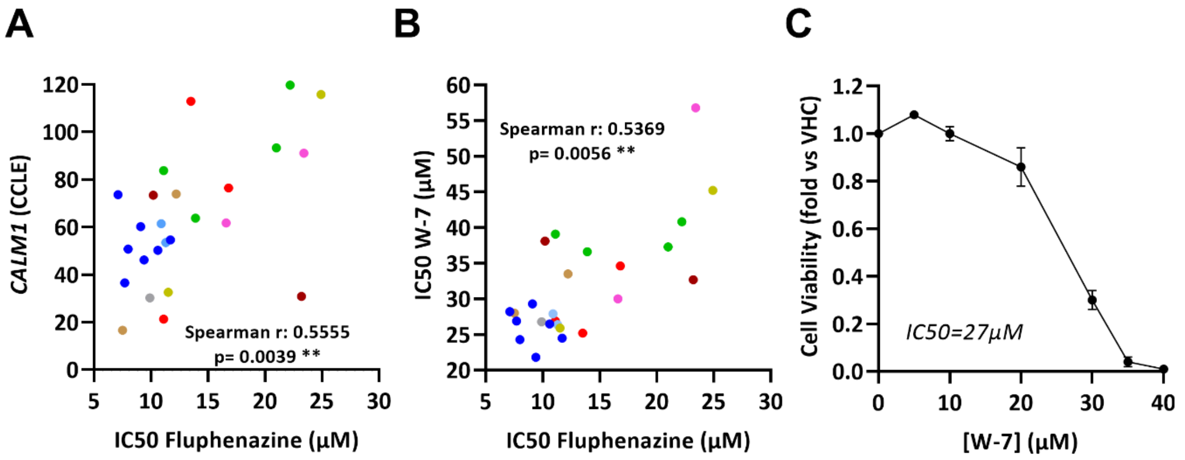

D

Spearman test (r , P value)	IC50 CWHM-974	IC50 Fluphenazine
CALM1	**0.6506 ; 0.0004 *****	**0.5555 ; 0.0039 ****
CALM2	0.04851 ; 0.8179 ns	0.03616 ; 0.8637 ns
CALM3	0.1648 ; 0.4312 ns	0.1066 ; 0.6122 ns

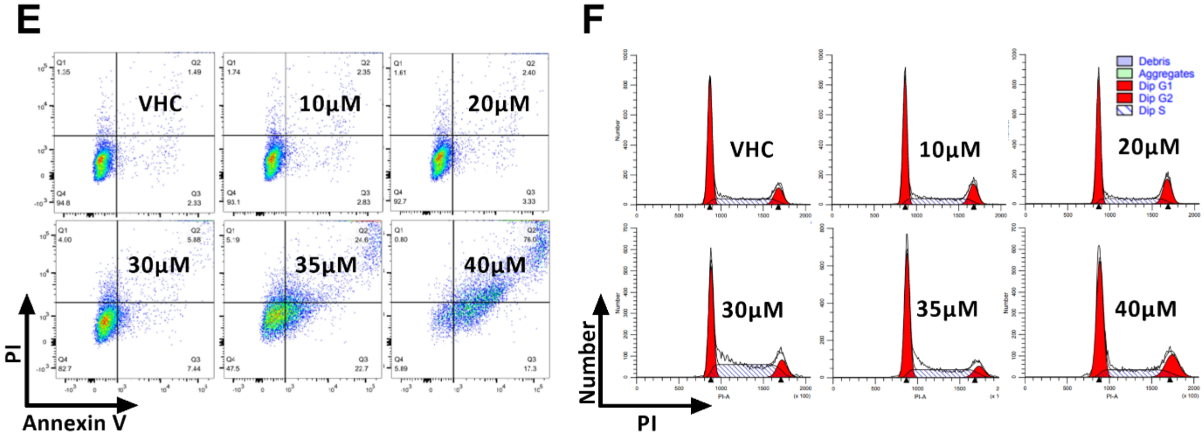

Supplementary Fig. S4.

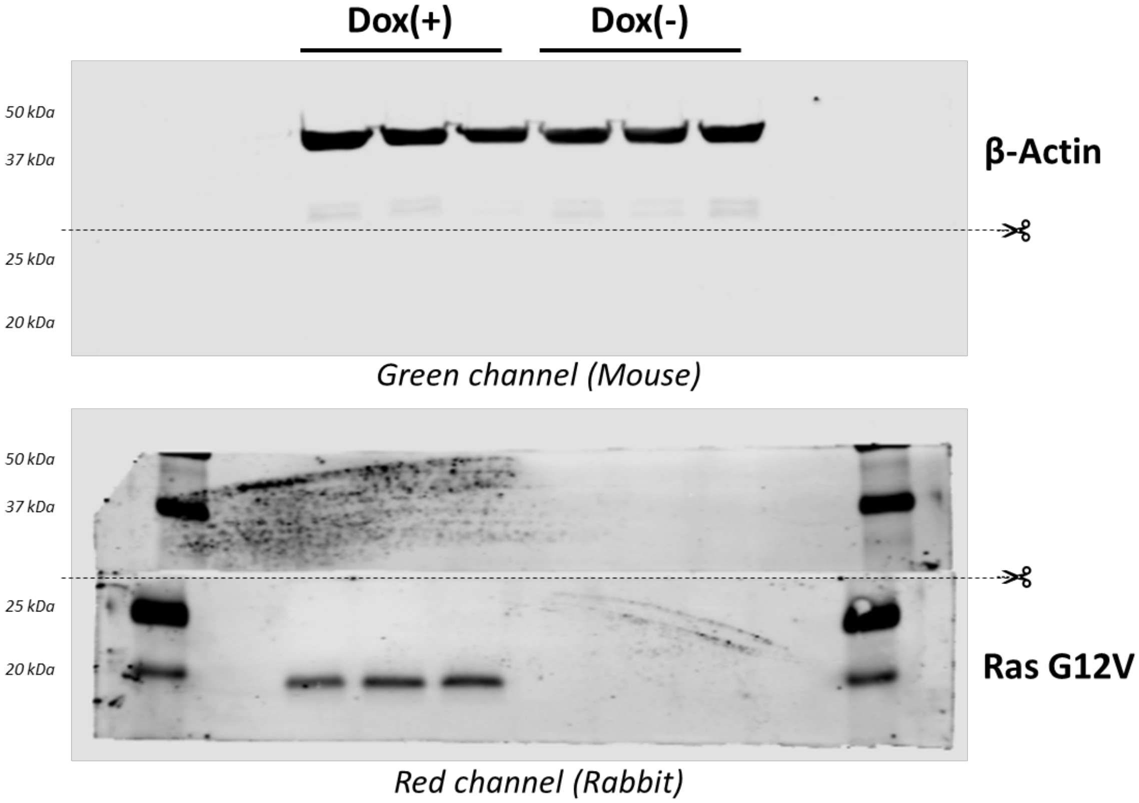

Supplementary Fig. S5.

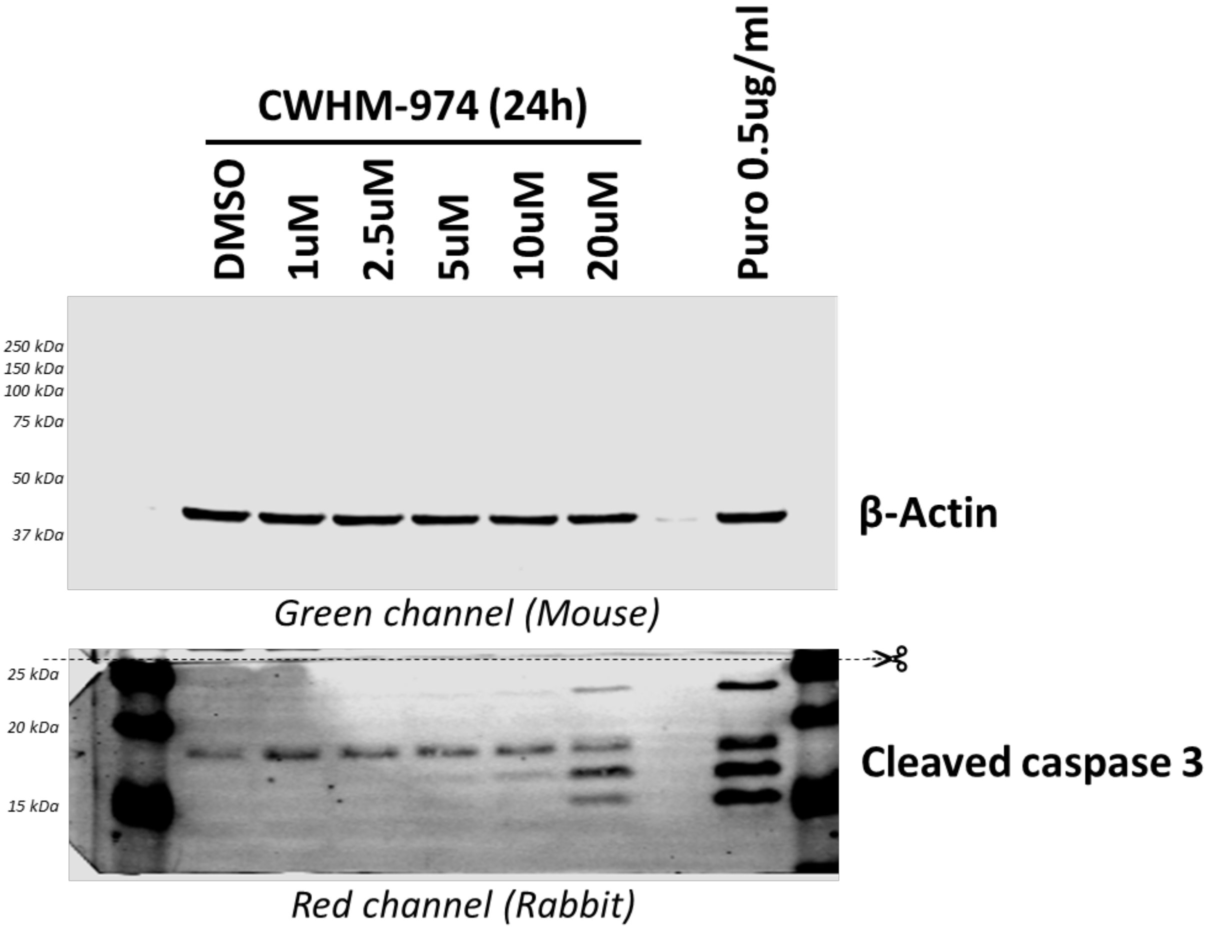

Supplementary Fig. S6.

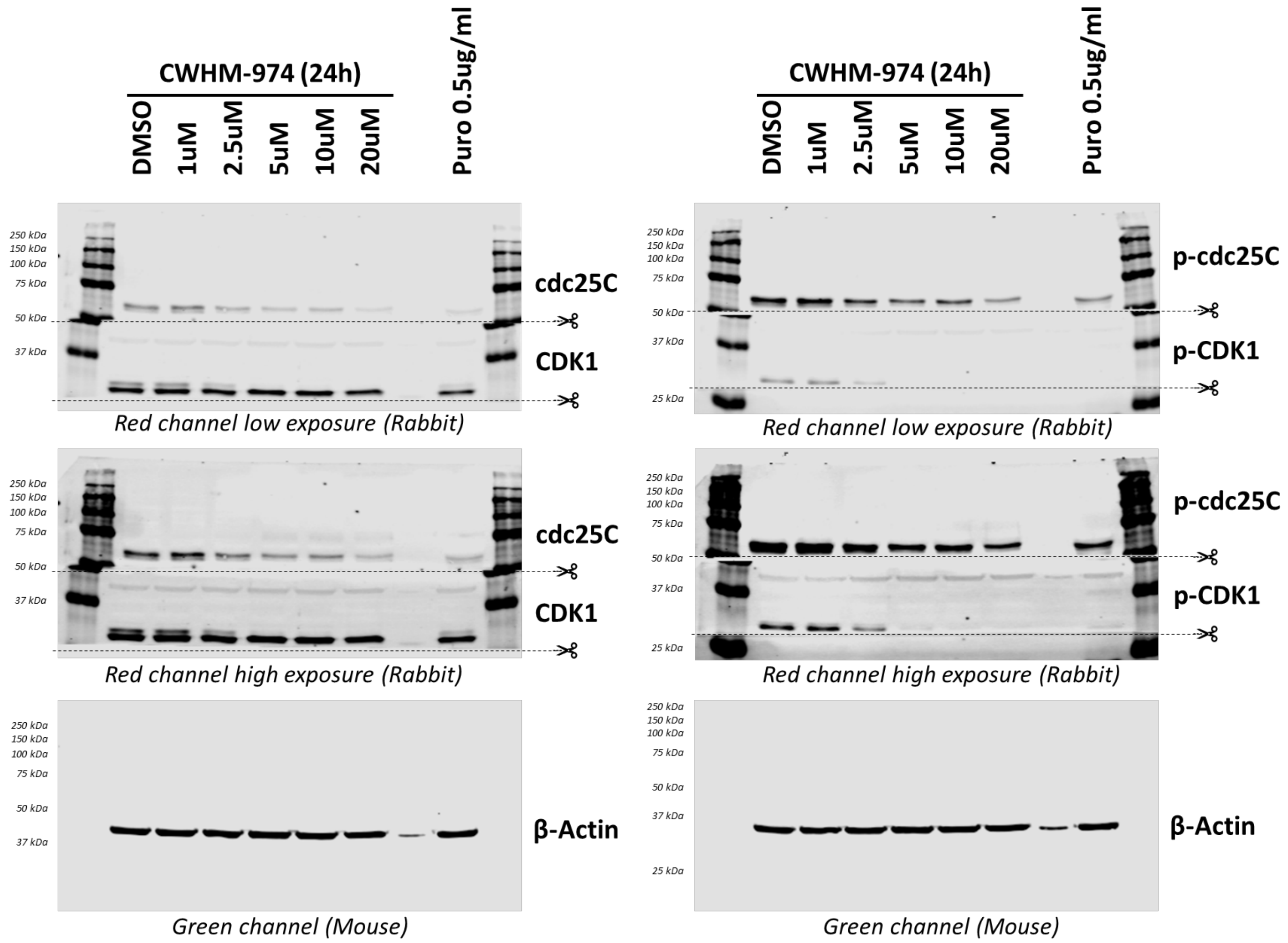

Supplement: Supplementary file 1 [file DataSheet_1.pdf]
